# Chloroform Death During Child-Birth

**Published:** 1911-11

**Authors:** F. G. Hodgson

**Affiliations:** Atlanta, Ga.


### CHLOROFORM DEATH DURING CHILD-BIRTH.

By F. G. Hodgson, M. D., Atlanta, Ga.

Mrs. P. P. Age about 24. Primipara. I was called to give chloroform for a forceps delivery. The woman had been having hard labor pains for some time. The doctor was all ready when I arrived, and the patient suffering great pain. So I proceeded to give the $CHCl_3$ without any preliminary examination. I had been taught in medical school and from other sources, that chloroform was a perfectly safe anaesthetic during labor. She took the $CHCl_3$ well. The doctor had some difficulty in getting the forceps on and asked me to help him. So I turned the chloroform over to the husband, washed up, went around, put on the forceps, pulled the head down onto the perineum. Then I turned the forceps over to the doctor in charge of the case and went back to take charge of the chloroform. The woman stopped breathing just before I got to her head and we were unable to make her breathe again. The child's head was out and delivered post-mortem.

This was the first death I had seen due to chloroform during child-birth, and being absolutely contrary to what I had been taught is my reason for reporting it. The patient was a stranger to the doctor in charge and to me, and we know nothing of her history in regard to previous heart trouble or to her pregnancy. She had the appearance of a normal, healthy woman. She was at no time very deeply under the influence of the anaesthetic.

---

### COMMUNICATION.

*Editor of the Atlanta Journal-Record of Medicine*:

I have read with interest Dr. Hansel Crenshaw's contribution, "The Classification of Pellagra," in the last issue of the Journal-Record. Dr. Crenshaw treats with a somewhat good-humored derision the claim of the dermatologist on pellagra. That this province of medicine is as legitimately entitled to a share of proprietorship in the disease equal to if not greater than that of other branches of medcine having points of contact with the disease, I shall endeavor to show. It is a significant fact that the disease from its most conspicuous sign received the name of pellagra, a bastard word from the union of the Italian *pelle,* the skin, and the Greek *agra,* rough; rough skin. This at once places it nosologically, in the same category with such admittedly dermatological preserves as erythema, eczema, lichen planus, leucoderma and a host of other skin affections named for their physical characters and in which one or more organs or systems of the body are concerned in their etiology or symptom-complex. Until the recent agitataion over pellagra in this country, the disease was given perfunctory notice in systematic works on the practice of medicine or was omitted altogether. Neurological text books are uniformly silent. On the other hand, dermatological treatises have all given the disease more or less extensive consideration. Crocker devoted six pages of his work on diseases to pellagra, Kaposi three, McCall Anderson four—and all of these were written at the time when pellagra was endemic to certain unimportant portions of Europe and was attracting a very limited amount of attention. These circumstances clearly indicate that pellagra by custom and precedent fell in the class of affections belonging to the field of dermatology. So much for the older or pre-American conception of Pellagra.

The present day notions of Pellagra have strengthened rather than weakened the dermatological claims upon the disease. It is generally admitted that the earlier the disease is recognized, the better the prognostic outlook. How is the disease recognized? The time-honored classification of pellagra would seem to supply the answer. The eruption of pellagra is distinctive, the gastro-intestinal and nervous systems taken alone are not. Camurri says, quoted in Marie's Pellagra, "The most important medical problem now occupying the pellagrologic field is without doubt that of establishing an early diagnosis. Without this it is impossible to arrest or ameliorate the harmful effect of pellagra which seem fatally inevitable." In the same work in connection with diagnosis occurs the remark that a tentative diagnosis must frequently await the appearance of more conclusive symptoms, especially the erythema. Of the skin lesions and their value in diagnosis Merk says (ibid.) that these suffice for a diagnosis and that the cutaneous lesions of pellagra possess the same value in diagnosis as do those of scarlatina, measles, variola and varicella in the respective diagnosis of these diseases. Grave doubt has often been expressed by competent observers of the existence of pellagra without eruption, pellagra sine pellagra, maintaining that this disease like syphilis always shows cutaneous reactions at some time during its course.

Diagnosis of pellagra without eruption is then tentative, unsatisfying, inconclusive and perhaps unjustifiable. Gastro-intestinal symptoms are common to many affections, the nervous and mental phases of the disease belong to established classifications, the skin symptoms alone are *sui generis,* distinctive and set doubt at naught.

If the diagnosis is of the first importance and is based essentially upon the cutaneous signs, who then is the proper person to make the diagnosis? It is obviously the function of one whose eye and brain are trained to the analysis of skin lesions rather than to one whose training has not been of this character. Only yesterday I saw a young girl with the typical pellagrous eruption on the dorsa of the hands and upon the posterior aspect of the forearms. The tongue was bright red with prominent papillae. There was no other symptom of the disease. The eruption had been diagnosed as eczema, a dangerous error when one considers the importance of early diagnosis.

After the diagnosis is made and the disease recognized beyond doubt, in the matter of treatment is the dermatologist to be forced back upon his exclusive privilege of warts, moles and facial blemishes for lack of ability from experience to cope with anything larger or more serious? Contrast by way of rejoinder the free drafts upon materia medica and therapeutics required by the dermatologist for the successful management of the manifold connections of cutaneous disease with the rather thin armamentarium of the gastro-enterologist and the notoriously spare therapeutics of the neurologist. The truth is, as everybody knows, that there is no specific in the treatment of pellagra, results are discouraging and one physician whatever his affiliations is about as competent to treat the disease as another.

To recapitulate, the dermatologist's claim on pellagra is supported by precedent, by his superior training in differential diagnosis of skin lesions (taken in connection with the high importance of early diagnosis of pellagra), and by the fact that the requirements of his branch fit him for competence in the general management of the disease. The right of the dermatologist to treat syphilis has never been disputed, why then by a strong analogy, should it be questioned in pellagra?

BERNARD WOLFF.

Atlanta, Ga., Oct. 18, 1911.

---

### SURGICAL SUGGESTIONS.

---

In trachelorrhaphy care must be taken not to close the cervical canal at any point.

When tuberculous involvement of the Fallopian tubes is evident to the naked eye, pan-hysterectomy should be performed.

Simple perforation of the uterus during a curettage in an aseptic field requires no further treatment than a packing of gauze in the uterus.

A chronic suppuration in the middle ear may be due entirely to an adhesion near the floor and internal wall, forming a pocket in which pus may lodge.

Irrigating the throat with ice water from a fountain syringe will frequently relieve the congestion and give great comfort in cases of acute follicular tonsillitis.

Both ether and chloroform anaesthesia have a hemolytic effect, which is followed by a compensatory polycythemia. It is followed also by 30 per cent. increase in the leucocytes, which begins during anaesthesia and lasts for about 24 hours. Leucocytosis is also induced by saline infusions and purgation.—American Journal of Surgery.

